# Stigma, an important source of dissatisfaction of health workers in HIV response in Vietnam: a qualitative study

**DOI:** 10.1186/1472-6963-12-474

**Published:** 2012-12-21

**Authors:** Ha Nguyen Pham, Myroslava Protsiv, Mattias Larsson, Hien Thi Ho, Daniel H de Vries, Anna Thorson

**Affiliations:** 1 Department of Public Health Sciences, Division of Global Health (IHCAR), Karolinska Institutet, Stockholm, Sweden; 2 Hanoi Medical University, Hanoi, Vietnam; 3 Oxford University Clinical Research Unit, Hanoi, Vietnam; 4 Hanoi School of Public Health, Hanoi, Vietnam; 5 Department of Anthropology & Sociology, University of Amsterdam, Amsterdam, The Netherlands

## Abstract

**Background:**

Like in many other low- and middle-income countries, the recent development of an HIV epidemic in Vietnam has led to a growing need for prevention, treatment, care, and support services for people living with HIV (PLHIV). This puts greater demands on the national HIV services, primarily on health workers, which increases the importance of their job satisfaction and working conditions. This study describes health worker perceptions and explores the factors that influence job satisfaction and dissatisfaction of health personnel working on the HIV response in Vietnam. Spector’s job satisfaction model was used as the theoretical framework for the study design and analysis.

**Methods:**

The study employed a qualitative design with 7 focus group discussions and 15 semi-structured interviews with health workers, purposively selected from national and provincial organizations responsible for HIV services in 5 cities and provinces in Vietnam. Data were analyzed using a hybrid approach of theory-driven and data-driven coding and theme development using qualitative analysis software.

**Results:**

HIV services are perceived by Vietnamese health workers as having both positive and negative aspects. Factors related to job satisfaction included training opportunities, social recognition, and meaningful tasks. Factors related to job dissatisfaction included unsatisfactory compensation, lack of positive feedback and support from supervisors, work-related stress from a heavy workload, fear of infection, and HIV-related stigma because of association with PLHIV. An adjusted Spector’s model of job satisfaction for HIV service health workers was developed from these results.

**Conclusion:**

This study confirmed the relationship between stigmatization of PLHIV and stigma experienced by staff because of association with PLHIV from families, colleagues, and society. The experiencing stigma results in additional work-related stress, low self-esteem, poor views of their profession, and lower income. The study shows the importance of actions to improve staff job satisfaction such as pay raises, supportive supervision, stress management, stigma reduction and workplace safety. Immediate actions could be the provision of more information; education and communication in mass media to improve the public image of HIV services, as well as improvement of workplace safety, therefore making health workers feel that their work is valued and safe.

## Background

In recent years the perception of health workers has changed from one of almost being completely neglected to recognition as a crucial part of health systems [1]. The health workforce needs to be viewed not as a resource, but rather as a partner in delivering health services. It is thus very important to move one step further by understanding how health workers experience their work in relation to the HIV epidemic [2]. The increasing prevalence of HIV infection every year has led to increased demand for services for people living with HIV (PLHIV) that in turn increases the burden on the health system and the workloads of health workers in HIV service organizations. Response to the HIV epidemic is currently one of the first priorities in public health. Shortages of human resources have been cited as the major barrier for increasing HIV services in low- and middle-income countries [3-5]. The World Health Organization (WHO) has suggested that countries should improve the recruitment process, make the most of their existing workforces, and manage the migration of healthcare personnel to other countries. Therefore, appropriate remuneration, financial and non-financial incentives, promotion of lifelong learning, and creating an enabling work environment are needed [1]. Given the pivotal role that health workers play to achieve good service, it is important to understand what motivates them and the extent to which they are satisfied by the organizations for which they work.

Job satisfaction describes how people feel about their jobs—whether they like or dislike their jobs [6]. Job dissatisfaction has been cited as the primary reason for high turnover [6,7] and absenteeism [6,8], which in turn poses a threat to organizations’ capacities to provide quality service and meet the needs of customers [9]. Studies have shown that dissatisfied employees are more likely to quit their jobs or be absent than satisfied emplo-yees [7,10]. Therefore, increasing job satisfaction and organizational commitment are potentially good strategies for reducing absenteeism and turnover intentions [10]. However, job satisfaction is not the same as motivation, although they might be clearly linked. In the work context, motivation can be defined as an individual’s degree of willingness to exert and maintain an effort towards organizational goals [11]. Motivation can be divided into two types: intrinsic and extrinsic motivation. Intrinsic motivation exists within the individual and refers to motivation that is driven by an interest or enjoyment in the task itself [12,13]. Extrinsic motivation refers to the performance of an activity to attain an outcome [12,13]. A study by Goetz et al. showed that intrinsic motivators have the most positive impact on job satisfaction of dentists in Germany [14], while Sicsic et al. wanted to send a cautionary message on the potential negative effects of increasing extrinsic moti-vation among French general practitioners through their pay-for-performance policy [13]. There are several theories on job satisfaction. Hackman and Oldham argued that five core job characteristics (skill variety, task identity, task significance, autonomy, and feedback) affect three psychological states (experienced meaningfulness, responsibility for outcomes, and knowledge of actual results) that, in turn, influence job performance, job satisfaction, motivation, and turnover [6,15]. Herzberg pointed out that certain factors cause job satisfaction, while a separate set of factors cause dissatisfaction [14,16]. Spector’s job satisfaction survey assesses job satisfaction through nine job facets: pay, promotion, supervision, fringe benefits, contingent rewards, operating procedures, co-workers, nature of work, and communication [6].

Vietnam, with a population of approximately 86 million [17], has a concentrated HIV epidemic, with the highest prevalence among people who use drugs (13%), female sex workers (3%), and men who have sex with men (17%) [18]. The adult HIV prevalence (ages 15–49) was 0.45% in 2011 [19]. The first HIV case was reported in 1990 and it is estimated that there will be up to around 263,000 PLHIV by 2015 [18]. The need for HIV prevention, care, and treatment is high, but insufficient quantity and limited quality of health staff, together with poor incentives and rapid turnover among staff in HIV service organizations, have been cited as the main problems in Vietnam [18]. As an attempt to identify measures to enhance the work performance and retention of the health workforce, this study addresses two research questions: (1) how health workers perceive their work on HIV response in Vietnam; and (2) what factors influence their job satisfaction and dissatisfaction. Spector’s job satisfaction model was used as a theoretical framework in study design and analyses while other theories of jobs satisfaction, specifically which of Maslow and Herzberg, were also used for comparing and discussing the study’s main findings.

## Methods

### Study design

This study employed a qualitative design with focus group discussions and semi-structured interviews. The study used a hybrid approach of theory-driven and data-driven coding and theme development for data analysis [20-22].

### Settings

This study was conducted in five cities and provinces in Vietnam, namely Hanoi, Quang Ninh, Khanh Hoa, Ho Chi Minh City, and Can Tho, which represent the northern, central, and southern parts of the country. These areas have a relatively high HIV prevalence and large numbers of donor-funded projects.

### Study participants

Participants were purposively selected from national and provincial HIV service organizations. The participants constituted a diverse group of middle and senior managers including medical professionals, social workers, and representatives of civil organizations.

### Data collection

Seven focus group discussions (FGD) with 80 participants and 15 individual semi-structured interviews were conducted throughout 2009. Guides for group discussions and interviews were developed based on the Human Resources for Health Action Framework jointly formulated by the Global Health Workforce Alliance, USAID, and the WHO [23]. The guides included topics to be covered and a set of suggested questions on human resource management systems, policy, partnerships, leadership, finance, and education. The group discussions included participants from the same level of management. Each discussion lasted for 2.5 hours. The group discussions focused on issues such as factors influencing the work environment and conditions (employee relations, workplace safety, salaries, allowances, and incentives), major sources of job satisfaction and dissatisfaction, training, and career development. Individual interviews were arranged for higher-ranking managers who were not available for group discussions, or whose participation might have prevented other participants (i.e., their subordinates) from talking freely about sensitive issues. Each interview lasted 1 hour. Interviewees were asked about their opinions and thoughts on policies that affect human resource management, performance appraisal and supervision, and leadership development. Two professional facilitators and two secretaries were engaged in data collection. The group discussions and interviews were recorded, and written notes were taken by secretaries. After each discussion and interview, the secretaries and facilitators listened to the tapes and expanded and/or corrected the notes. Then the tapes were transcribed, translated into English, and imported into NVivo 8 software (QSR International Pty Ltd, Victoria, Australia) which was used to perform the data analysis.

### Data analysis

The study used a hybrid approach of theory-driven and data-driven coding and theme development for data analysis [20-22] with a previously established list of theoretical categories derived from Spector’s Job Satisfaction Survey (JSS) [6]. According to Boyatzis, in theory-driven code development, the researcher begins with a theory of what occurs and then formulates the signals, indicators, or evidence that would support this theory. The elements of the code are derived from the hypotheses or elements of the theory [20]. Data-driven codes are constructed inductively from the raw information. They appear with the words and syntax of the raw information [20]. Fereday and Muir-Cochrane used this approach in their study and described it as a hybrid approach of inductive and deductive coding and theme development [21]. Similarly, Atkins et al. analyzed data initially using qualitative content analysis, and then organized the resulting categories under the constructs of the Normalization Process Model [22]. In the present study, the process of data coding and analysis was conducted in several steps. Initially, the transcripts were read several times to achieve familiarity with the content. Next, the meaningful units were detected in the text, labeled, and coded. The unit of analysis was a complete expressed idea or thought, which normally consisted of one or several sentences. Similar codes were constantly compared with each other in the process and some codes were revised. Moreover, we constantly returned to the context of the separate references to ensure that the codes were compatible with the raw data (in Vietnamese). Next, the codes were grouped under sub-categories, which were compared and agreed upon among the authors. Using the JSS as a framework, the sub-categories were organized into nine categories that corresponded to the nine factors previously listed. Themes were developed through more abstract analysis as described by Boyatzis [20] and Graneheim and Lundman [24]. This hybrid approach provided us with an existing theoretical framework that helped us organize the data and simultaneously allowed us to apply a data-driven coding process to catch important context-related factors that could not be accounted for in the original theory. Credibility was established thorough inter-coder checking, which required the first author to compare the coding in Vietnamese with that of the second author who later coded the data in English. The preliminary results were also checked in a similar fashion. The collaboration of researchers with different backgrounds ensured a triangulation of the researchers’ perspectives, to a certain extent.

### Ethical consideration

In 2009, the Vietnam Administration of HIV/AIDS Control (VAAC) gave permission to IntraHealth International to carry out the Capacity Project Assessment of Human Resources Needs for Management and Coordination of HIV Prevention, Treatment, Care and Support Programs in Vietnam. The project was conducted according to the Vietnamese rules and regulations on research and studies, which included a review of all aspects (e.g., operational or ethical) by the VAAC and IntraHealth International. Participants provided informed consent via oral agreement before the interviews and group discussions. Note-takers did not record the names of the participants and the findings of the interviews and discussions were not shared outside the research team.

## Results

In the process of analysis, we grouped different aspects of the job as described by participants into categories corresponding to the facets of job satisfaction, the categories together constituting an overall assessment of staff’s job satisfaction. Because the analysis did not exclude other important factors that could be found in the data, we added new categories originally not included in Spector’s JSS. Work on HIV services is perceived by health workers as having both satisfactory and unsatisfactory aspects. Factors causing job satisfaction included training opportunities, social recognition, and doing meaningful tasks. Factors causing job dissatisfaction included unsatisfactory compensation, lack of positive feedbacks and supports from supervisors, work-related stress, fear of infection, and stigmatization of the profession because of association with PLHIV. Table 1 list these categories and factors and identifies them according to the positive or negative connection that the participants made to the overall feeling of job satisfaction.

**Table 1:** Categories and factors causing job satisfaction and dissatisfaction

**Categories**	**Factors causing job satisfaction (+) and dissatisfaction (−)**
Pay	(−) Unsatisfactory salaries
	(−) Limited opportunities for additional income generation
	(−) Difference in pay between payroll and project staff
Promotion	(+) Recognition leading to promotion
	(−) Job insecurity in project staff
Supervision	(−) Inadequate supervision measures
	(−) Lack of understanding of supervisory tools
	(−) Lack of positive feedback from supervisors
	(−) Rewards tied to annual appraisal: weak, poorly implemented
Fringe benefits	(+) Adequate number of training opportunities
	(−) Uneven distribution of training opportunities
	(−) Uneven distribution of benefits
	(−) Disincentives
Contingent rewards	(+) Recognition in society
	(+) Intrinsic motivation
	(+) Meaningful tasks
Operating procedures	(−) Personnel policies: excluding some categories of staff, not enough transparency
Nature of work	(+) Many job opportunities
	(−) Uneven distribution of job opportunities
	(−) Increasing workload
	(−) Risk of being infected through contact with PLHIV
	(−) Work-related stress
Communication	(−) Outdated, inefficient ways of communication
	(−) Low capacity in IT for communication
Stigma	(−) Attitudes towards key populations at risk in society
	(−) Stigmatization of PLHIV
	(−) Stigmatization of profession because of association with PLHIV

### Factors causing dissatisfaction

#### Unsatisfactory compensation

Pay and monetary benefit was one of the factors most frequently mentioned in the interviews and group discussions affecting job satisfaction. This theme reflects the feeling shared by the participants of being poorly compensated in terms of salaries, incentives, and benefits. While the compensation discussion is a natural part of the pay and fringe benefits category, the less expected phenomenon is the participants’ emphasis that work hazards (exposure to HIV and other infections) should result in additional remuneration. Dissatisfaction with compensation affects employees’ overall attitudes about their jobs. Participants reported that their current level of pay was low and, therefore, did not meet their needs to support themselves and their families.

*People left because salaries were not enough to live on. I can still sit here because I can rely on my husband; otherwise, my own salary would not be enough to buy medicine [if I get sick].* (National agency FGD participant)

Current salaries for health workers are perceived as insufficient to satisfy basic needs. Therefore, staff are forced to look for alternative sources of income. Many of the participants are entitled to various allowances when attending conferences, seminars, workshops; working on outreach activities, or short-term consultancies, especially for the international donor–funded projects. The additional income-generating opportunities common among other health workers, such as those at private practices, are not available to HIV service health workers because of the poverty of patients with HIV-related illness and the aforementioned stigma. Furthermore, non-HIV-infected patients are reluctant to see doctors who work with these infected patients.

*Doctors have difficulties to work in private clinics. For example, patients do not want to come to see them for ear-nose-and-throat treatment. HIV is stigmatized, so normal people do not come for medical check-ups and treatment with these doctors.* (Southern Province FGD participant)

#### Uneven distribution of fringe benefits

Because of the low salaries, fringe benefits are an essential part of employees’ income that supplements their payroll earnings. Receiving lower or no benefits is considered a major cause for dissatisfaction among staff. Nevertheless, the distribution of the benefits is unequal among staff categories. Some professions, not necessarily the ones with the highest wages, receive small or no benefits at all. The distribution is uneven between different health facilities.

*Allowances for workers at communes and villages are around VND120,000, which is just enough to fill up the gasoline tank really; thus, it is hard to require them to concentrate only on their works. I myself feel so sad thinking about this. There is no insurance system for them, as they are not full-time but social workers. The staff working for provinces or districts in contrast have official payroll positions.* (Southern Province FGD participant)

A participant commented on treatment versus prevention:

*Health workers working with HIV-positive patients in hospitals receive allowance of 40% [sectoral hazardous allowance], low but something, while preventive health workers receive nothing. Preventive care is declared the priority of health care, but for preventive care remuneration is considered as a minor problem (laughed).* (Southern Province FGD participant)

#### Disincentives

Disincentives are practices that some managers or leaders perform with negative consequences to payment or benefits of the staff and which have a discouraging effect on staff morale. Here we have two different examples of disincentives as lowering staff’s salary:

*Some donor projects can provide high salaries but the Vietnamese managers decide the lower levels saying that [it is needed] to keep the levels close to government salaries and that such high levels are not sustainable when projects phase out. The projects suggest 4 million but we just agree with 2 million. Some people said their salaries are even lower than that of the government because the Vietnamese managers lower them.* (Southern Province FGD participant)

*I see that some projects do not pay at all. One example is our project; there was no remuneration while they should pay us at least some kind of allowances for our working time for the project.* (Southern Province FGD participant)

The practice of benefits sharing can be observed in some workplaces. This involves dividing the allowance that some part of staff receives among the total number of workers. The reason behind this action is managers’ understanding of fairness and equality.

*The regulation on hazardous allowance is that health workers who work directly with patients with HIV-related illness receive an additional 40% salary allowance. This is a good incentive. However, this allowance is not applied to all staff, and if I am the doctor who cares for patients, when I am on duty or annual leave, someone else will take care of the patients. As allowance is given to a certain number of staff, the department has to make adjustments. For example, the department has allowances for 4 doctors and 2 nurses out of 10 staff, so the department has to redistribute allowances to all in a harmonious way.* (National agency FGD participant)

Both practices are possible because of a lack of policy enforcements to protect workers against managers abusing their power in terms of redistribution of budgets intended for staff compensation. The practices are perceived as discouraging and harmful to the staff.

#### Weak supervision

This theme reflects the perception that there is a lack of supervision for the employees, which affects their feelings about their jobs. The current supervisory practice focuses on task accomplishment or failure. Therefore, achievements remain underappreciated and unrecognized, which can be frustrating for the staff. From the interviews and focus groups, we conclude that the feedback mechanism between the managers and subordinates is rather weak.

*The important thing is to train people who conduct supervision. I have been trained so the work is somehow less difficult for me, but my staff have limited supervision skills. Even in medical university, they do not teach about supervision. People conduct supervision based on their own assessments. We have to do supervision step-by-step because people must change their views that supervision is a fault-finding task.* (Northern Province interviewee)

The quotation above is an example of the recognized need for supervision and the current lack of supervisory training at medical schools and elsewhere. In addition to the lack of supervisory capacity, other staff members have a limited understanding of supervision. Junior staff tend to be afraid of strict supervisory measures that they refer to as “inspection” and try to resist them, while the supervisors rely on their own judgments about how supervision should be conducted. In the interviews, lack of positive feedback from supervisors was related to the current ineffective system of monitoring tasks, which fails to record and recognize employees’ good performance and achievements. This unsystematic way of monitoring performance is perceived as unfair and might be related to dissatisfaction.

*If our performance in this is good, nothing will happen, but in case performance is not so good, it will be reflected in our evaluation.* (Southern Province FGD participant)

*Yes. It will be taken into account if we fail and nobody will comment if we do it well.* (Southern Province FGD participant)

Overall, participants perceived themselves as poorly informed, which could be seen as a cause for dissatisfaction.

#### Work-related stress

Participants expressed concerns about the increasing workload because of the growing number of HIV infections, which results in an increasing demand for treatment, care, and support. They also expressed their concerns about the increasing number of patients and the growing workload with the number of staff remaining the same. The increasing workload puts higher demands on staff, which results in higher turnover, and ultimately increases the workload of the remaining staff.

*We lack sufficient staff. It is difficult to recruit more doctors and nurses. Newly recruited staff stayed with us for a few months then moved to other departments or to other hospitals where working conditions are better; there are fewer risks of infections and less stress.* (Southern Province FGD participant)

A feeling of being overloaded at work is known to influence an employee’s attitude towards their job. However, in our findings, job dissatisfaction with the growing work overload could be further increased by the feeling that no effort is being taken by the management to improve the situation. The fear of infection combined with stigmatizing attitudes toward the patients was also related to the participants’ levels of stress. Fear of transmission should not be a significant issue for employees, assuming they are well informed about the routes of transmission and prevention measures.

*The staff that do counseling and make contacts with infected people, they do not get any extra allowance. In prisons, prisoners with HIV infection are numerous. Many of our staff also are infected with tuberculosis. We proposed that doctors and nurses who work directly with these prisoners should be given some incentives.* (National agency FGD participant)

Participants talked about monetary incentives with respect to high risk, while neglecting the issue of work safety. However, there is currently no clear understanding of policies about occupational accidents involving health wor-kers. Participants were very concerned about whether they would be compensated if they became infected at work.

*They are most afraid of being infected. There are no regulations on compensation for staff that are infected. They do not know whether they will receive compensation for occupational accidents.* (Northern Province interviewee)

#### HIV-related stigma

One of the most important factors contributing to staff dissatisfaction was stigma.

Because of the concentrated pattern of the HIV epidemic in Vietnam, infected individuals are likely to be drug users and sex workers. These groups are highly stigmatized in Vietnamese society and have been referred to as “social evils” by the government. Some employees seem to share these attitudes and, therefore, resist direct contact with PLHIV whenever possible.

*The biggest constraint is that staff may not want to have direct contacts with infected people, most of whom have relations with social evils.* (National agency interviewee)

The combination of prejudice against PLHIV and the perceived high risk of infection are responsible for stigma among health workers. Work on HIV response is a stigmatized profession, and the stigma toward health workers originates primarily from attitudes toward the key at-risk populations. Furthermore, there is a great deal of prejudice from colleagues in other health domains.

*Because of the prejudice toward PLHIV, there is also prejudice towards the health workers who care for them. I even said to our management that we treat patients and we are put in the same group with them. Doctors who are assigned to HIV treatment are also considered as lower grade than doctors in other departments.* (Southern Province FGD participant)

Consequently, the attitudes of other employees and family members contribute to the stigmatization of health workers involved in HIV services.

### Factors causing satisfaction

#### Training opportunities

Opportunity for training was a top reason for staff satisfaction. There are plenty of training opportunities; however, they are usually only available to mid-level staff at the provincial and central levels. The managers recognize the importance of training for staff and support them in using these opportunities.

*HIV services have many activities and projects. It is a good place for young staff to try their capacities. It is obvious that their capacities improved a lot when working here. They have many opportunities for learning, both in the country and overseas.* (National agency FGD participant)

However, training opportunities are unevenly distributed in the same way, as are fringe benefits. Staff in district and rural areas have few opportunities for development and training, which make those workplaces even less attractive.

*The human resources for the whole sector need to be further enhanced. At the grassroots level, it is difficult to provide training, whereas working requirements are harder. Therefore, many doctors do not want to work at the grassroots level*. (National agency interviewee)

#### Intrinsic motivation

This section describes which aspects of the participants’ jobs they find motivating. First, the staff enjoy their work because of its humanitarian nature and their sympathy and willingness to help others.

*Most people are dedicated to social development and human values, so they are happy to carry out these activities.* (Southern Province FGD participant)

And

*After some time of working here, I also have found the work interesting. I feel sympathetic toward the patients, if you think of them as your relatives, you will have more sympathy.* (Southern Province FGD participant)

#### Meaningful tasks

The work on HIV care and treatment was related to higher morale and awareness of work quality. Participants clearly feel the importance of their work. They receive meaningful tasks and see that they can make an impact with their inputs. They feel responsible with high satisfaction from the work, and the earning of respect from others.

*We love our job. We devote our efforts to work to gain effective outputs. Some international organizations have offered me jobs with good salaries, which would be great for my family, but these jobs cannot contribute as much as the current one. Here, my work will have broader influence.* (National agency FGD participant)

#### Social recognition

Being awarded with titles such as “Best Employee” was considered important for several reasons. First, it is hono-rable to receive such recognition from the organization, as exemplified by a participant’s views:

*If they work well, the organization will give rewards, possibly in cash. There are also many honorable titles to be awarded, for example, Advanced Employee, or Best Employee.* (National agency interviewee)

The titles can also be viewed as a positive experience for employees because they come along with a monetary reward as well as a demonstration of recognition from the management.

*I myself am keen on this work because it is relevant to my expertise and interests. Many people work because of their love and responsibility to the work. In addition, your work is compensated and respected by the others.* (Central Province interviewee)

The second reason why titles are perceived as important is that they provide the possibility to be recognized by the management, which might lead to a promotion. Overall, we conclude that participants value rewards such as monetary benefits and recognition from society. However, participants consider the system of monitoring employee performance—the basis of distributing awards—unfair. For job satisfaction, this means that rewards and recognition positively influence employees’ attitudes towards their jobs. However, poor implementation of annual awards is a reason for dissatisfaction.

We generated five themes related to job satisfaction (Table 2).

**Table 2:** Themes of job satisfaction

**Themes**	**Categories**	**Factors**
*Unsatisfactory compensation*	Pay	Unsatisfactory salaries
		Limited opportunities for additional income
	Fringe benefits	Uneven distribution of fringe benefits
		Disincentives
		Uneven distribution of training opportunities
*Weak supervision*	Supervision	Lack of positive feedback from supervisors
		Lack of understanding of supervisory tools
		Inadequate supervision measures
	Contingent rewards	Annual staff appraisal and rewards: unfair, poorly implemented
	Communication	Outdated, inefficient ways of communicating
*Work-related stress*	Nature of work	Increasing workload
		Perceived risk of being infected through contact with PLHIV
*HIV-related stigma*	Nature of work	Negative attitudes towards key populations at risk in society
		Stigmatization of PLHIV
		Stigmatization of professionals because of association with PLHIV
*Motivation factors*	Nature of work	Training opportunities
	Fringe benefits	Intrinsic motivation
	Contingent rewards	Meaningful tasks
		Social recognition

## Discussion

We identified factors causing job satisfaction and dissatisfaction of HIV service health workers in Vietnam. Here we look at the relationships among these factors and compare them with theories of job satisfaction of Maslow, Herzberg, and Spector.

### Unsatisfactory compensation

A theme of unsatisfactory compensation emerged when discussing job dissatisfaction with the participants. This theme includes low salaries, limited additional income-generating opportunities, differences in pay between project staff and government payroll positions, and the uneven distribution of benefits. Pay plays an important role in job satisfaction theories. According to Herzberg, pay belongs to the hygiene factor group that does not provide positive satisfaction, but results in dissatisfaction when absent [16]. In Maslow’s theory, pay helps individuals meet their physiological needs [25]. Spector points out that the correlation between pay and job satisfaction tends to be surprisingly small, which suggests that pay itself is not a strong factor in job satisfaction; however, pay fairness can be a very important factor [6]. Similarly, a study by Songstad et al. of health workers in a rural district in Tanzania showed that unfairness in salary level, allocation of allowances, promotions, access to training, and upgrading reduced staff motivation, affecting their work performance [26]. The participants in the present study were concerned that people in the same job earned more money because they were involved in projects that were funded by international organizations. Salary and benefits are taken for granted, but could be critical if they are not competitive with others’ [27].

### Weak supervision

Participants mentioned dissatisfaction related to organizational factors, particularly the lack of positive feedback and poor rewards from supervisors. Furthermore, participants viewed supervision as an act of control. Feeling neglected by supervisors had a strong demotivating effect. Apart from more supervision, participants desired more instruction and needs-oriented supervision, which would provide direct and timely feedback. Herzberg argued that weak supervision can lead to dissatisfaction [16]. Supervision is one of the factors in Spector’s JSS [6]. WHO strongly argues for good quality supervision, noting that “supervision that is supportive and helps to solve specific problems can improve performance, job satisfaction and motivation” [1].

### Work-related stress

Other studies have confirmed the findings that work on HIV services is stressful [28-31]. The causes of stress are workload [28-30], fear of infection [31-33], dealing with drug users, and little support from colleagues [31,34]. Workload has been perceived by health workers as having both mental and physical aspects; e.g., the level of difficulty and the amount of work they must do [6]. The increasing workload associated with the growing number of HIV cases was one of the major concerns of health workers in this study. Raviola et al. found that high workloads caused stress, low self-efficacy, fatigue, and frustration in staff [29]. Kalichman et al. [35] found that work-related stressors could be divided into workplace-related and patient-care-related stress, the latter is responsible for most stressful events. Their study found that job stress originated from the demanding work of taking care of patients, but some parts of this stress were because of the fear of infection transmission and social stigma. This assumption is supported by previous studies that identified fear of transmission as a common stressor [29-31,36]. Some participants in the present study mentioned HIV as a serious occupational hazard, while others recognized exposure to tuberculosis as a more potent threat. Previous studies have shown that “irrational fears” of contracting infections resulted in stress and higher perception of risk among health workers [30,31]; however, this fear did not appear to result in compliance with safety measures [30]. In the present study participants mentioned, that fear of infection was an issue for young and inexperienced employees in particular, which was confirmed by another study based in Vietnam [37]. Fear of infection has also been identified as the main factor contributing to the ‘reluctance’ of colleagues from other departments to collaborate with HIV service workers to provide care and treatment for PLHIV [30,38,39]. Another study found that health workers did not have a regular supply of disposable gloves and antiretroviral drugs for post-accident treatment, which resulted in higher levels of stress [29]. With better access to preventive measures, health workers perceive themselves to be better protected and more comfortable at work [33]. Therefore, the WHO recommends comprehensive infection-control strategies and procedures including standard precautions [40]. According to Maslow, safety is the second fundamental need after physiological needs and includes health and well-being [25]. According to Herzberg’s theory, safety belongs to the hygiene factor group that does not provide positive satisfaction, but results in dissatisfaction when it is absent [16]. Spector found that high job stress and burnout levels are associated with greater intention to leave a job [6]. Stress can also result in behavioral reactions (e.g., quitting a job), physical reactions (e.g., hypertension), and psychological reactions (e.g., frustration) [6]. Li et al. provided evidence that institutional support is important to promote a positive psychological state and to prevent burnout and departure from the workforce [33].

### HIV-related stigma

UNAIDS defines HIV-related stigma as “a process of devaluation of people either living with or associated with HIV” [41]. Our study identified three different categories related to stigma based on the source and target of stigmatization: i) stigma toward key populations at risks in society, ii) stigmatization of patients with HIV-related illnesses, and iii) stigma experienced by health workers originating from society, colleagues, and families. The third category of stigma is recognized as “associated stigma” [42], or “perceived stigma” that includes both stigma health workers create and the stigma they experience as a result of their work [43]. Consistent with other studies, we show that health workers are influenced by common negative attitudes associated with drug users and sex workers [30,44,45]. As part of society, health workers are understandably influenced by societal norms, attitudes [33], and prejudices [30]. Therefore, we conclude that stigma towards this profession has a negative impact on employees’ perception of their work, and ultimately their job satisfaction. Several studies have highlighted considerable reluctance in significant proportions of health staff that would prefer not to work with HIV-positive patients if given the choice [30-32,46]. Fear of infection is a significant contributing factor to this reluctance [30-32,45]. Similarly, other studies in Vietnam have found a reluctance to provide services [36,37,45]. In the present study, “social evils” and HIV as a punishment for practicing socially unacceptable behaviors were attitudes that came up in the interviews and discussion groups. The “social evils” attitude is unique to the Vietnamese context and refers to sex work and drug use. “Social evils” and HIV have been closely related since the beginning of the epidemic because the main government priority was to reduce the spread of the infection by combating sex work and drug use [47]. In a study on stigma and discrimination toward HIV-positive patients in health facilities in Vietnam, Khuat et al. conceptualized, i) HIV fear-based stigma; i.e., fear of casual transmission and related stigmatizing attitudes led health workers to treat these patients differently, and ii) HIV value-based stigma because of negative values / social judgments and associations among HIV and certain behaviors and groups, such as sex workers and injection drug users. They show that fear-based combined with social stigma reduction intervention was more effective than an intervention primarily focused on fear alone [45]. Other studies have described similar attitudes [36,44,46]. Therefore, we conclude that such attitudes are common in Vietnamese and other contexts and are shared by many other people, including health workers. Stigma has been found to have a significant negative influence on the level of job satisfaction [43,48]. A study in China showed that health workers who observed a higher level of discrimination against PLHIV in society were more likely to report being a victim of stigmatization and discrimination. This suggests that social norms and environment play an important role in forming these attitudes towards health workers. This finding also implies that stigma reduction is essential to promoting a higher quality of care [33]. A study in five African countries identified perceived stigma as the strongest predictor of job dissatisfaction leading to nurses’ intending to migrate to other countries [43]. Health workers in South Africa reported feeling a lack of professional respect, were labeled as incompetent by other (non-HIV) doctors, and lacked recognition from the public for the ‘good and stressful job’ that they do, thus “creating an impetus to leave the HIV work” [49]. Therefore, stigma may contribute to the health workforce shortage in HIV service organizations, which suggests that strategies are necessary to improve retention [50] and job satisfaction [43]. Job satisfaction is more commonly explained by factors such as salaries, working conditions, availability of supplies, and opportunities for advancement. The present study demonstrates that job dissatisfaction is partly due to stigma. The study suggests that stigma reduction among health workers and society as a whole will improve job satisfaction. Immediate actions could be the provision of more information; education and communication in mass media to improve the public image of HIV services, as well as improvement of work safety, therefore making health workers working in the area feel that their work is valued and safe.

### Motivation factors

This study revealed some interesting findings regarding the nature of work within HIV service organizations, suggesting that this is potentially satisfying work. Participants were motivated to work in this area because of its humanitarian nature, their sympathy for others, their eagerness to help, and the encouragement they received from society. They were internally motivated to do this work because it brings them pleasure. This motivation is conceptualized as intrinsic motivation [12-14]. Previous studies partially confirm this finding and add that positive feelings of health workers are related to their ability to help and care for stigmatized people [30]. These feelings reduce the negative effects of stress and burnout [51]. Maslow argued that after the physiological and safety needs, it is necessary to obtain feelings of love and belonging [25].

We found that participants value contingent rewards such as appreciation and recognition from the organization and society. According to Herzberg, social recognition is one of the motivators that give people satisfaction [14,16]. Some studies have also attempted to identify the rewards that health workers receive in caring for PLHIV [30,51]. The positive outcomes reported by nurses included the ability to help, interactions with patients, and admiration for patients’ courage [30]. Breault and Polifroni conducted interviews with nurses and identified the following rewarding outcomes: making patients comfortable, seeing a patient go home, and helping a patient die with dignity. They obtained satisfaction from providing what they believed was non-judgmental care to stigmatized people [52]. Health workers in a study by Nashman et al. said that providing comfort and support was satisfying. Educating patients, staff, and others was also a major source of satisfaction and self-gratification (i.e., “knowing I am doing well”) [30]. Nurses interviewed by Reutter and Northcott said their work was enjoyable and worthwhile because of the relationships they developed with their patients [53]. Nurses also received feedbacks from patients and their families which assured that their work was valued [30].

All participants in the present study mentioned training opportunities as an important motivator. Training enables health workers to aspire to more demanding duties and positions and to achieve their professional advancement goals. Participants also appreciate the annual rewards and positive feedback from supervisors. Titles such as “Best Employee” are viewed as positive experiences because they come along with monetary rewards and provide the possibility of promotion. This motivation is conceptualized as extrinsic motivation [12-14]. The study by Goetz et al. on job satisfaction with dentists in Germany showed that both intrinsic and extrinsic factors are important in determining the perception of job satisfaction, while the presence of internal-motivational factors such as opportunity to use abilities and recognition for work have the most positive impact on job satisfaction [14]. This feeling of satisfaction is supported by concepts such as esteem, self-actualization, and self-transcendence in Maslow’s theory [25]. Similarly, growth, learning, and advancement are considered motivators that lead to satisfaction in Herzberg’s motivation-hygiene theory. Sicsic et al., examine the relationships between intrinsic and extrinsic motivators among French general practitioners in the pay-for-performance model. They reported the potential side effects of the model with the erosion of intrinsic motivation by extrinsic rewards [13].

### Stigma as a main dissatisfaction factor

As a result of analysis, we identified themes that correspond to causes of employees’ dissatisfaction in HIV services: poor compensation, lack of positive feedbacks and rewards from supervisors. Moreover, we were able to find factors previously not included in theoretical frameworks such as HIV-related stigma, work hazards (exposure to HIV and tuberculosis), work-related stress, and increasing workload because of the development of the HIV epidemic in Vietnam. Among these findings, it is hard to distinguish general working conditions in the health sector and those specific to work in HIV services. The study demonstrated the high value of training opportunities, but not equal access to those causing dissatisfaction. Similarly, we found that compensation of health workers is insufficient to cover health workers’ basic needs, although they do value permanent employment from income stability. Moreover, participants reported that annual awards seem to be an important reward. We were also able to identify some factors, which were not included in the list of theoretical categories based on Spector’s JSS. These new factors that were later included in the adjusted model might be related to the context and setting of the study—work in public health facilities in Vietnam, the peculiarities of HIV services, or a combination of both. We found that HIV-related stigma is manifested as a resistance of health workers to have direct contact with PLHIV. We took into account the social role of the doctors in Vietnamese culture, which is a very respectful and honorable profession. We predicted that stigmatization of employees of HIV service organizations by association with PLHIV can have a very negative impact on the employees’ views of their job and profession.

### Adjusted Spector’s job satisfaction model

**Figure 1:**
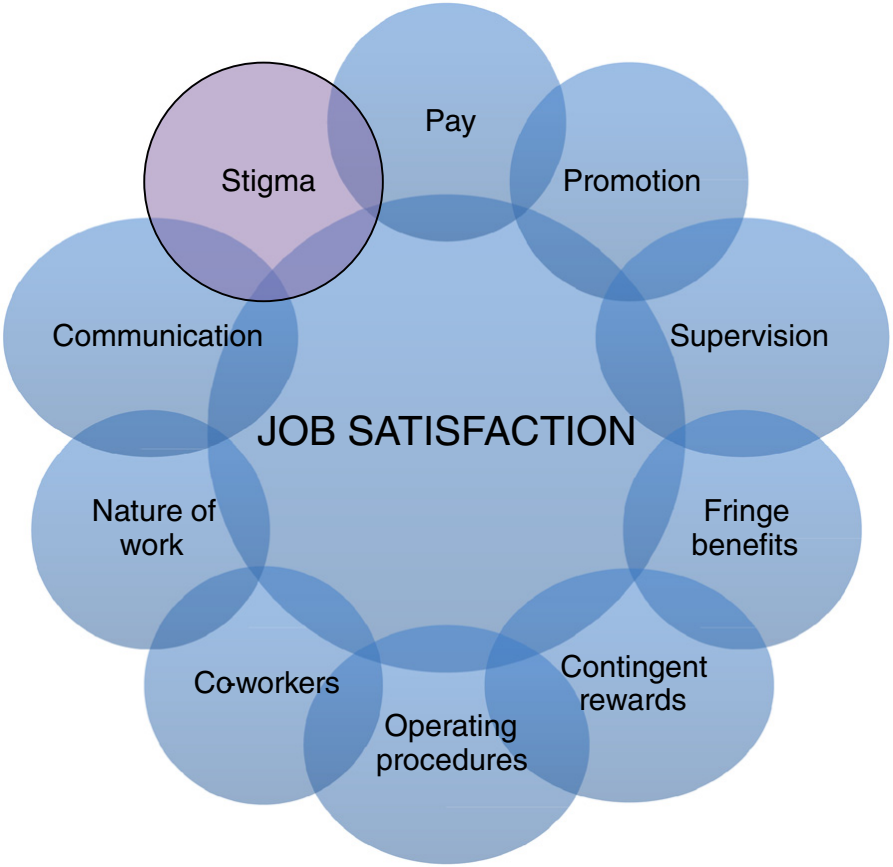
Adjusted Spector’s job satisfaction model of health workers in HIV service organizations.

This study suggests an adjusted Spector’s model of the job satisfaction of HIV service health workers in Vietnam (Figure 1).

Although presented separately in this model, the components are closely related to each other and often work together to influence employee job satisfaction. Each component could become a target for intervention. For example, actions could be taken to improve work safety and to mitigate stigma and discrimination in hospitals.

### Limitations

This study has limitations concerning the research design and sample. In terms of the design, this qualitative study could not provide an overall score of job satisfaction or the weight of each factor. The purposively selected sample was not necessarily representative of health workers in Vietnam. It is also important to note that the study participants might have used the interviews and focus group discussions as an opportunity to raise their concerns; therefore, data on the positive factors might have been less forthcoming than data on the negative factors.

## Conclusion

We found job satisfaction of HIV service health workers in Vietnam to be affected by poor compensation; lack of positive feedback and support from supervisors. Work within HIV service organizations might put unique demands on employees. We found that job satisfaction is influenced by stigma, some working conditions experienced as work hazards, high work-related stress, and increasing workload, which were not accounted for in the theoretical framework. We confirmed the relationship between stigmatization of PLHIV and stigma experienced by staff because of association with PLHIV from their families, colleagues, and society in general, and stigma impact on staff job satisfaction. We hypothesized that experiencing stigma results in additional work-related stress, low self-esteem, poor views of their profession, and lower income. The work of caring and supporting patients with HIV-related illness was considered demanding in terms of high levels of stress. We suggest that manifestations of stigma, such as unrealistically high perceptions of occupational risk, result in higher levels of stress in staff. We show that high workloads are an important issue that needs to be resolved. Insufficient staffing needs to be addressed to maximize retention and increase job satisfaction.

### Implications for practice and further research

The study findings suggest there is a need to improve working conditions of health workers in HIV service organizations to ensure motivated human resources are in place to meet the growing demand for HIV services in Vietnam. The study findings regarding the influence of stigma on staff job satisfaction suggest the need for further study of how prevalent stigma is in health workers within the HIV response field and what the mechanisms of its influence are. In practice, stigma reduction interventions should be targeted at staff members, their families, and colleagues.

Management of organizations such as health departments, provincial HIV/AIDS control centres, hospitals, preventive health centres, and HIV service organizations at the central level should target their efforts to improving working conditions, primarily with regard to staffing levels and workload management, improving access and information about work safety, and preventing work-related stress and poor outcomes in employees.

Opportunities for further research might include conducting longitudinal or interventional studies to investigate how changes in the factors discussed in this article influence job satisfaction.

## Abbreviations

AIDS: Acquired Immunodeficiency Syndrome; FGD: Focus Group Discussion; FSW: Female Sex Workers; HIV: Human Immunodeficiency Virus; IDU: Injection Drug User; JSS: Job Satisfaction Survey; PLHIV: People Living with HIV; VAAC: Vietnam Administration of HIV/AIDS Control; UNAIDS: Joint United Nations Programme on HIV/AIDS; USAID: United States Agency for International Development; WHO: World Health Organization.

## Competing interests

The authors declare that they have no competing interests.

## Authors’ contributions

PNH and AT conceptualized the study. DV designed the methodology for the Capacity Project on Assessment of Human Resources Needs for Management and Coordination of HIV/AIDS Prevention, Treatment, Care and Support Programs in Vietnam, which provided data for this study, and continued to be involved in manuscript writing. PNH, MP, ML, HTH, and DV all contributed to data analysis and editing the manuscript. PNH and MP prepared the main manuscript. All authors read and approved the final manuscript.

## Authors’ information

PNH is a PhD student at Karolinska Institutet and is employed at WHO Vietnam. MP has a Master of Public Health degree and is employed at Karolinska Institutet. ML has a PhD in public health and is employed at Oxford University Clinical Research Unit in Hanoi, Vietnam. HTH has a PhD in public health and is a lecturer at Hanoi School of Public Health. DV has a PhD in anthropology and is employed at the Centre for Social Science and Global Health, University of Amsterdam. AT is a medical doctor, has a PhD in global health, and is employed as an associate professor at Karolinska Institutet.

## Pre-publication history

The pre-publication history for this paper can be accessed here:

http://www.biomedcentral.com/1472-6963/12/474/prepub
